# Epidemiological Changes of Respiratory Syncytial Virus (RSV) Infections in Israel

**DOI:** 10.1371/journal.pone.0090515

**Published:** 2014-03-03

**Authors:** Shira Hirsh, Musa Hindiyeh, Liat Kolet, Liora Regev, Hilda Sherbany, Karnit Yaary, Ella Mendelson, Michal Mandelboim

**Affiliations:** 1 The Mina & Everard Goodman Faculty of Life Sciences, Bar-Ilan University, Ramat-Gan, Israel; 2 Central Virology Laboratory, Ministry of Health, Chaim Sheba Medical Center, Ramat-Gan, Israel; 3 Department of Epidemiology and Preventive Medicine, School of Public Health, Sackler Faculty of Medicine, Tel-Aviv University, Tel-Aviv, Israel; Alberta Provincial Laboratory for Public Health/University of Alberta, Canada

## Abstract

RSV is the leading cause of lower respiratory-tract infections in infants and therefore demands in-depth epidemiological characterization. We investigated here the distribution of RSV types in Israel between the years 2005–2012. Clinical samples were collected from 11,018 patients hospitalized due to respiratory illnesses and were evaluated for the presence of various respiratory viruses, including RSV A and RSV B. Until 2008, each year was characterized by the presence of one dominant type of RSV. However, from 2008, both RSV A and B types were detected at significant levels, particularly among infants aged 0–2 years. Furthermore, significant changes in the RSV A and RSV B subtypes circulating in Israel since 2008 were observed. Finally, we demonstrate that, irrespectively of the changes observed in RSV epidemiology, when the pandemic H1N1pdm09 influenza virus appeared in 2009, RSV infections were delayed and were detected when infection with H1N1pdm09 had declined.

## Introduction

Respiratory syncytial virus (RSV) is a pneumovirus belonging to the *Paramyxoviridae* family. It is a non-segmented, negative-strand RNA virus that expresses 11 proteins [1]. The virus infects the ciliated airway epithelial cells [2] and the infection leads to rapid destruction of these cells, making RSV the most common cause of bronchiolitis and pneumonia in children below the age of 2 [3]. The clinical symptoms that follow RSV infection include fever, rhinorrhea, cough, wheezing [4] and possibility of acute otitis media [5]. Infection with RSV is very common and the majority of children become infected within the first two years of their life [6]. Thus, study of RSV epidemiological behavior, particularly in young infants, is essential.

RSV is divided into two types, A and B [7], [8], based on antigenic differences that exist in their glycoproteins G and F [9], [10]. While the two groups circulate independently, group A often tends to be more dominant [11]. A number of studies were carried out to test whether RSV A or RSV B infections differs in their clinical outcome [12], [13], but no significant differences were found.

The various RSV A and RSV B types are further categorized into subtypes [14] based on the variable domain of the attachment G protein [15]. The differences among the various RSV subtypes are located mainly in the ectodomain of the G protein, which share only 44% amino acid sequence identity between the two subgroups, as compared to 83% identity in the trans-membrane and cytoplasmic domains [1], [10], [16].

Here, we investigated the epidemiological features of RSV infections in Israel from 2005 until 2012. Surprisingly, we observed changes in the type (RSV A and RSV B) and subtypes of RSV infection since 2008. In addition, we noted that the pandemic H1N1pdm09 influenza virus delayed the infection with RSV.

## Materials and Methods

### Ethics

The work described here is a retrospective study performed on anonymous patient respiratory samples that were analyzed for the presence of different common respiratory viruses such as RSV, hMPV, adenoviruses and influenza viruses, as part of the routine tests performed at Sheba Medical Center. No extra samples were obtained for this research. Informed consent was not required. The institutional review board (IRB) of the Sheba Medical Center approved this research (Helsinki Number 4982-07-SMC).

### Patient samples

Respiratory clinical samples (nasopharyngeal swabs or aspirates) were collected from 11,018 patients hospitalized at Sheba Medical Center, Israel, due to respiratory illnesses during the May 2005 to May 2012 winter seasons and tested for the presence of the above mentioned viruses.

### Clinical parameters

The clinical characteristics recorded in medical files of all patients studied here were also evaluated. The patients suffered from shortage of breath, fever, cough, rhinorrhea, dyspnea, lack of appetite, vomiting, diarrhea and sore throat.

### Viral analysis and sequencing

Patient samples were tested either by Rapid Diagnostic test for RSV detection (Coris Bioconcept, Belgium) or by extraction of viral RNA. The extraction of viral RNA was performed with either the High Pure viral RNA extraction kit (Roche Diagnostics GmbH, Mannheim, Germany) or the NucliSENS easyMAG (BioMerieux, France). Real-time reverse transcription-PCR (rRT-PCR) was performed to determine the RSV type (A or B) [17].

The tests were performed using multiplex (simultaneous amplification of more than one target sequence in a single reaction). In 2005–2011 the multiplex reaction contained RSV A and RSV B. In 2011–12 the multiplex reaction contained FluA, FluB, H1N1pdm and RSV (A+B together) [18], [19]. Reactions were performed using TaqMan Chemistry on the ABI 7500 instrument. For the RNA rRT-PCR assays, the Ambion Ag-Path master mix (Life Technologies, USA) was used according to the manufacturer's instructions.

### Phylogenetic trees

To establish a phylogenetic tree, viral RNA was extracted from 96 randomly selected samples (4.7% of all samples). The method used to generate the phylogenetic trees was previously described [20]. The specific primers used were:

RSV A: Forward – 5′-GTACCYTGCAGCATATGCA-3′, Reverse 5′-CAAMTCCATTGTTATTTGCC-3′.

RSV B: Forward 5′-ATGATTWYCAYTTTGAAGTGTTC-3′, Reverse 5′-GAATAACTAAGCATRTGA-3′

The 471 bp RSV A PCR products and 484 bp RSV B PCR products were purified using the Qiagen® High Pure PCR Product Purification Kit (Roche® Diagnostics GmbH, Mannheim, Germany) and sequenced using the ABI PRISM Dye Deoxy Terminator cycle sequencing kit (Applied Biosystems, Foster City, CA). Reaction mixtures were analyzed on the Applied Biosystems model 3100 DNA automatic sequencing systems.

The Sequencher® 5.0 program (Gencodes Corporation, Ann Arbor, MI) was used to compare the nucleotide sequences. Phylogenetic trees were prepared by nearest neighbor joint analysis using Clustal × with 1000 bootstraps, and trees were visualized using TreeView or NJ plot software. Phylogenetic trees were prepared using 50 RSV A sequences and 36 RSV B sequences. Sequences were deposited in the GeneBank (GenBank accession numbers: KF981452 -KF981469).

### Statistical analysis

The chi-test was applied to test the dependency/relationship between two variables. The t-test was applied to determine if two sets of data were significantly different from each other. A p value <0.05 was considered statistically significant. In the phylogenetic trees, bootstrap values of >70% are shown at the branch nodes.

## Results

### Distribution of RSV A and B types in Israel

Due to the changes we noted in the RSV types, while testing for the presence of various respiratory viruses we decided to fully characterize the RSV types that circulated in hospitalized Israeli patients between the years 2005 and 2012. To this end, samples obtained from 11,018 patients between the years 2005–2012 were re-examined for the presence of RSV A, RSV B, hMPV, adenoviruses, and influenza viruses and from 2009 and on, for the pandemic H1N1pdm09 influenza virus as well. The samples included 557 patients hospitalized from July 2005 to June 2006, 617 patients hospitalized in 2006–2007, 558 hospitalized patients in 2007–2008, 903 hospitalized patients in 2008–2009, 1,403 hospitalized patients in 2009–2010, 4,151 hospitalized patients in 2010–2011 and 2,829 hospitalized patients in 2011–12 (Table 1).

**Table 1:** Patient samples.

		#Positive (%)		
year	#specimens	#RSV-A	#RSV-B	total
**2005–6**	557	77 (78.6)	21(21.6)	230 (39.9)
**2006–7**	617	14 (17.1)	68 (82.9)	186 (33.1)
**2007–8**	558	80 (83.6)	16 (16.7)	164 (29.4)
**2008–9**	903	79 (38.0)	128 (62.0)	207 (22.9)
**2009–10**	1403	139 (59.9)	95 (40.1)	240 (17.1)
**2010–11**	4151	324 (67.1)	159 (32.9)	691 (16.6)
**2011–12**	2829	143 (46.1)	167 (53.9)	448 (15.8)

Of the 11,018 samples tested, 2,167 (approximately 20%) were RSV-positive (Table 1). Until 2011 all samples were tested for the presence of RSV by using the rapid diagnostic test for RSV detection. Only half of the RSV-positive samples from seasons 2005–6 to 2007–8 were also tested for the RSV type, as the type (A or B) is not considered clinically insignificant. However, once a change in RSV epidemiology was noted in winter 2008–9 all samples were analyzed for the presence of RSV A versus RSV B (Table 1). The same analysis was performed in 2009–10. In 2011, the multiplex detection assays were introduced in the laboratory, enabling simultaneous testing for several viruses, including the pandemic H1N1pdm09 influenza virus. Because in the years following the influenza pandemic many patients were hospitalized due to suspected illness with the pandemic H1N1pdm09 influenza virus, more samples were tested in 2010–2012 for the presence of RSV (Table 1).

**Figure 1:**
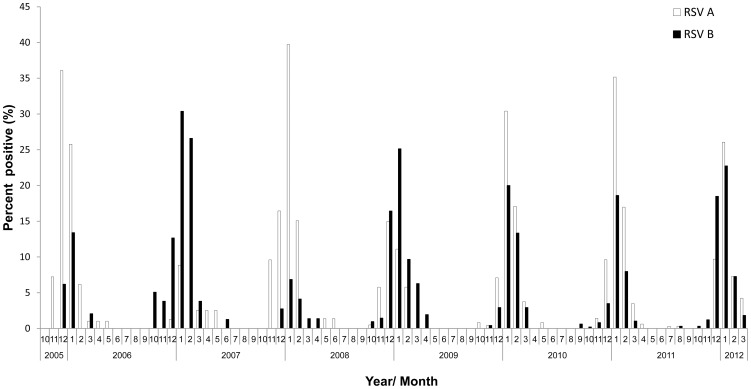
Monthly distribution of RSV A and RSV B infections in Israel. Summary of the percent monthly distribution of hospitalized RSV A- versus RSV B-infected patients, from 2005–2012. RSV type was determined by qRT-PCR. In each year, the total number of positive cases was set to be 100%.

Initially, the RSV type to first appear around October/November of the 2005–2006, 2006–2007 and 2007–2008 winter seasons was the one to become the dominant type of the season (Figure 1). In contrast, from 2008, no particular dominant strain was detected; both RSV strains were detected in relatively high percentages throughout the winter season (Figure 1).

**Figure 2:**
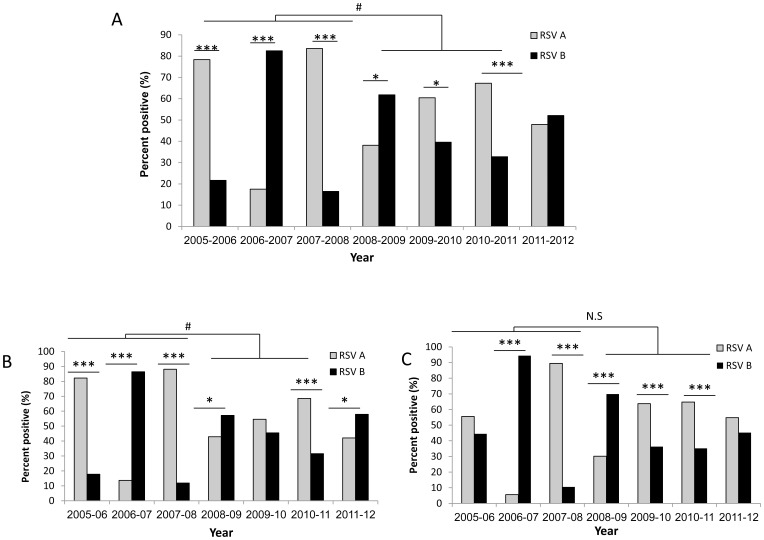
Yearly distribution of RSV A and RSV B types by age. Summary of the percent of RSV A or RSV B infection in the entire population (A), in infants (age of 0–2 years) (B) and in older patients (>2 years of age) (C). RSV A or B types were determined by qRT-PCR. In each season, the total number of positive cases was set to be 100%. Chi test was used to compare between RSV A and B positive samples, ***p<0.0001, *p<0.005. T-test was used to compare the relations between RSV A and B in the years before and after 2008. #: p<0.05 for dominancy in the three years before 2008 versus dominancy in the three years after 2008. N.S-not significant.

As mentioned above, due to advancements in respiratory virus detection, the usage of multiplex reaction and the emergence of the pandemic swine influenza in 2009, more adult samples were analyzed for the presence of RSV from 2008 and on. We therefore suspected that the drift observed in RSV infection from 2008 might be because more adults were analyzed. Thus, the numbers and percentages of RSV-type infected patients in each year, were summarized for the entire population (Figure 2A), for infants between 0–2 years of age (Figure 2B) and for all other patients (Figure 2C). As can be seen, until 2008, when analyzing the entire population, each year was characterized by the presence of a single dominant RSV type. Starting from 2008–2009, a particular RSV type was still dominant, yet, the less dominant RSV type was still detected in significantly higher percentages (Figure 2A). Indeed, prior to 2008, the differences between RSV A and RSV B infection was significantly higher than the same differences calculated in the years following 2008 (p<0.05). In the 2011–2012 season, no dominant virus was present (Figure. 2A).

Importantly, the loss of dominancy was detected primarily in infants (Figure 2B), while in older patients, dominancy of a particular RSV strain was still detected even following 2008. The only year in which no dominant strain was detected in patients older than two years of age was 2011–12 (Figure 2C).

### Disease severity of RSV-A and RSV-B infections

**Figure 3:**
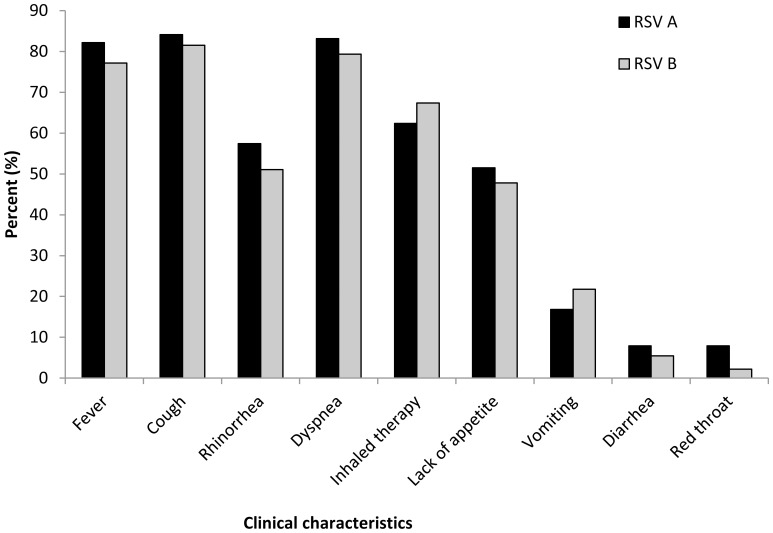
Clinical characteristics of the infected patients. The clinical symptoms presented by the patients hospitalized with RSV A or B from 2005–2012, are summarized. The presence of group A versus B RSV types was detected by qRT-PCR. The total number of all patients hospitalized between the years 2005 and 2012 was set to be 100%.

To test whether the loss of RSV dominancy observed from 2008 mirrored different clinical features, we examined the medical files of 725 patients that were infected RSV-A and of 618 patients infected with RSV B, where 56.4% and 58.5% of the examined cases for RSV A and RSV B, respectively, were infants under the age of 2. No significant differences were found between patients infected with either of the types, with respect to the presence of shortness of breath, fever, cough, rhinorrhea, dyspnea, lack of appetite, vomiting, diarrhea and sore throat (Figure 3).

### Changes in RSV subtypes from 2008 until 2012

**Figure 4:**
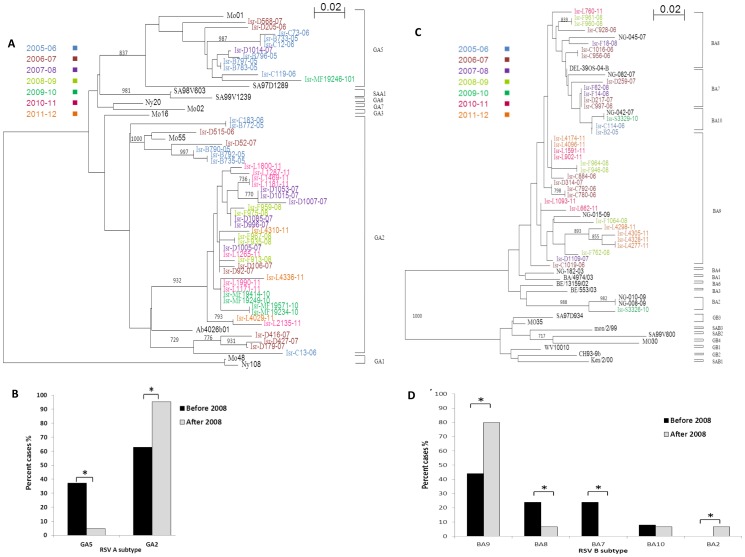
Phylogenetic analysis of the RSV A and RSV B subtypes. (A–D) Phylogenetic analysis of RSV A (A) and RSV B (C) infecting patients between the years 2005–2012. Strains are color-coded according to the season in which they appeared. The tree was generated by nearest neighbor joint analysis using Clustal × with 1000 bootstraps and visualized using TreeView or NJ plot software. Node values correspond to posterior probabilities. We used 50 out of 56 sequences to build the RSV A and 36 out of 43 sequences to build the RSV B tree in order to avoid identical branches. Bootstrap values of greater than 70% are shown at the branch nodes. (B and D). A summary of the various RSV A subtypes (B) and RSV B subtypes (D) that circulated in Israel are presented. *p<0.0003 before 2008 VS after 2008.

To investigate whether the partial loss of a single dominant RSV type, observed from 2008 and on, is associated with the elevated presence of different RSV subtypes, phylogenetic trees were generated to identify the subtypes of RSV viruses that were circulating in the country from 2005 to 2012. To this end, 96 samples were randomly selected (approximately 10 samples from each year, Figure 4). Only two identical sequences were included in the trees and sequences obtained more than twice were not included. The phylogenetic analysis revealed that most of the RSV A viruses were either GA5 or GA2 (Figure 4 A, B), findings which are consistent with other published studies [21]. All of the RSV B types clustered in the BA subtype (Figure 4 C, D). Until 2008, approximately 40% of the RSV A infections were due to RSV-GA5, while 60% were due to RSV-GA2. A dramatic change was noticed in 2008; from 2008 until 2012, the GA2 subtype was, by far, the most dominant RSV A subtype in the country (95.2%, Figure 4B). In parallel, until 2008, the BA7/8/9/10 subtypes co-circulated in the country, where BA9 comprised about 44% of the RSV B infections, BA7 and BA8 led to approximately 24% of the infections and BA10 accounted for only 10% of the hospitalized patients. In contrast, from 2008 and on, fewer patients were infected with BA8 and BA10 (∼6%), BA7 was not detected at all and BA2, a new subtype appeared. BA9 subtype became the dominant genotype, as 80% of the patients were infected with these viruses (Figure 4D).

The changes in the phylogenetic trees are not due to changes in the age distribution of samples, since most of the samples were obtained from children. In the RSV A phylogenic tree, only 2 out of 56 samples were taken from adult patients; one sample was obtained in 2007 from a 55-year-old patient and the other sample was obtained in 2010 from an 82-year-old patient (both samples had the GA2 subtype). In the phylogenic tree of RSV B, 4 out of the 43 samples were taken from older than 2 years old patients. One sample, obtained in 2010 from a 7-year-old patient, was found to be of the BA10 subtype. Two additional samples were taken in 2011 from a 72-year-old and a 4-year-old patient and were found to be of the BA9 subtype. Another BA9 sample was taken in 2007 from a 55-year-old patient.

### The pandemic H1N1pdm09 influenza virus delayed infection with RSV

**Figure 5:**
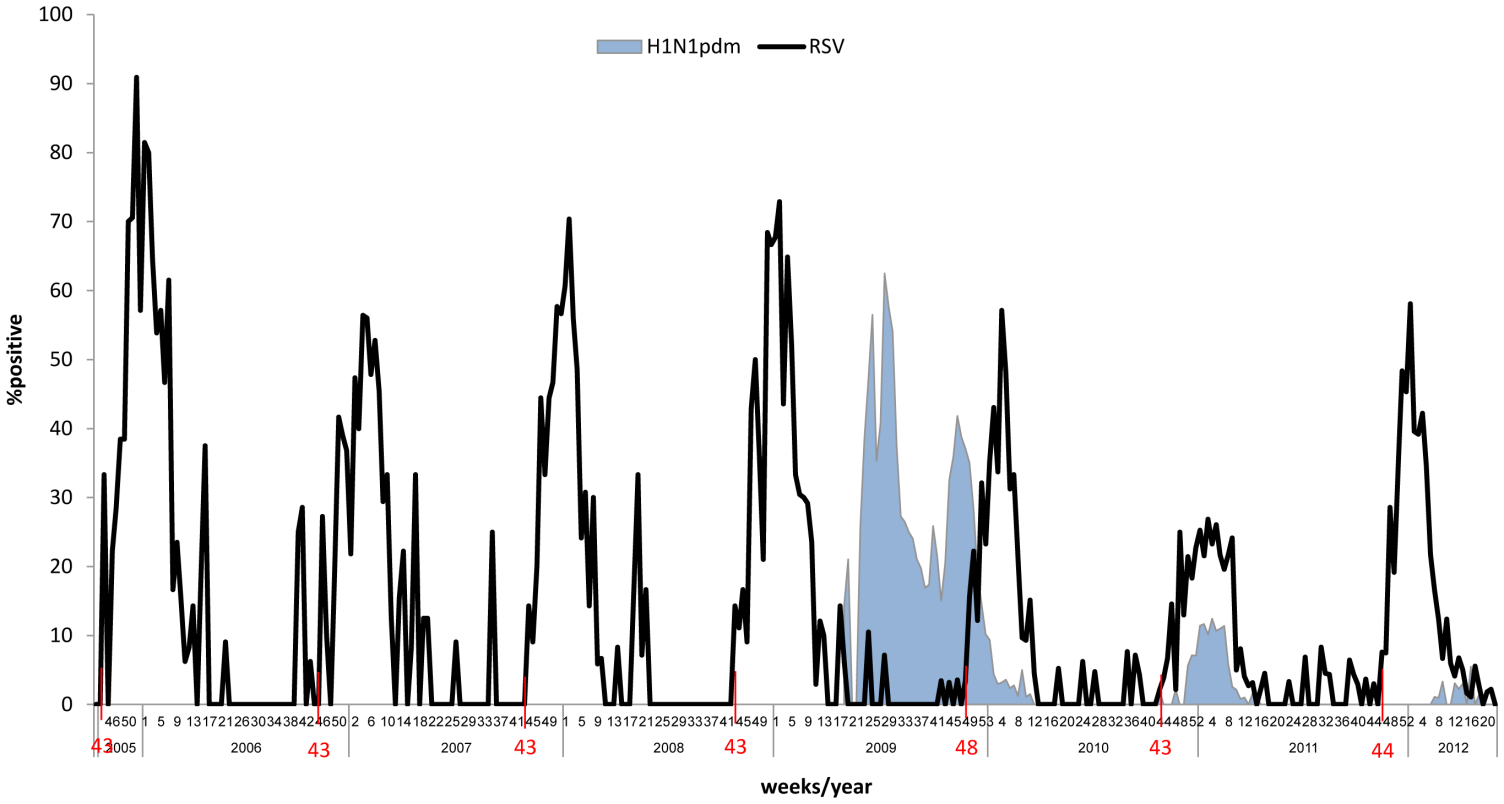
Delay of RSV infection following appearance of the pandemic H1N1pdm09 influenza virus. The weekly distribution of the percentages of patients hospitalized due to influenza-like symptoms and also found positive for infection with RSV, is presented. In each year, the week number of outbreak initiation is indicated.

In 2009, the pandemic H1N1pdm09 influenza virus reached many countries around the world, and first appeared in Israel at approximately week 17 of the same year. The pandemic swine influenza infection had two peaks and interestingly, the 2009 RSV infections were first noticed only around week 48, when the infection with the pandemic swine influenza began to decline (Figure 5). RSV infection rates declined between weeks 13–21 of the next year. In the following years, the onset of RSV infection was reset and infections first began in week 43, as usual (Figure 5). Interestingly, in 2010–11, infections with RSV were observed together with the pandemic swine influenza infection, and may reflect the conversion of the pandemic influenza virus from a pandemic virus to a regular seasonal influenza virus.

## Discussion

Although both RSV A and RSV B circulate in particular areas, typically only one type is responsible for each seasonal outbreak and will be the dominant type for several years before being replaced by the other [21], [22]. One possible explanation for these alterations is the development of specific immunity against a specific RSV type that is prevalent in the country at the preceding year. Indeed, we observed that a single virus strain dominated in winter seasons 2005–2008, yet, surprisingly, from 2008 and on, both RSV A and RSV B were present at significant levels. Importantly, the drifts in RSV dominancy were primarily observed in infants between 0–2 years of age, while in older patients, dominancy was still noticed, with the exception of one year.

Examination of the relationship between clinical characteristics and RSV types demonstrated no significant differences in patient's symptoms. Other several studies have examined the relationship between clinical severity and RSV types and subtypes. In most of these studies and in agreement with our results no differences in patients' symptoms between the RSV types were observed [10], [11], [13], [23]–[29]. In few studies, group A of RSV seemed to be associated with more severe clinical disease [30]–[35], while in few other studies group B infections have been reported to cause more severe disease [36], [37]. This inconsistency could be attributed to difference in study design and population, definition of disease severity, the distribution of RSV subtypes, etc.

In the current study, although the clinical symptoms did not changes over the years, different RSV subtypes were detected from 2008, concomitant with the partial loss of dominancy of the RSV types. These changes could not be attributed to the fact that more adult samples were tested from 2008 and on, because the samples that were evaluated in the phylogenetic trees were mostly obtained from children.

The changes in the RSV subtype observed from 2008 were detected for both the GA and the BA subtypes. The GA2 subtype was present in very high percentages, as observed in other geographic areas such as Europe, China and India. [38]–[40]. The continuous and predominant circulation of subtype GA2 demonstrate that this subtype is stable and has become epidemic in many countries. The dominant subtype of RSV B was BA, which contains a 60-nucleotide G-protein gene duplication, first appeared in Argentina in 1999. This observation also lies in agreement with other reports demonstrating that the BA subtype has become the most dominant subtype of RSV B globally [21], [39], [41], [42]. In Israel, five RSV B clusters were detected: BA2, BA7, BA8, BA9 and BA10, where the BA9 subtype became the dominant subtype in 2008.

Although the rapid global spread of BA viruses suggests that these viruses may have a selective advantage over other circulating viruses, no major outbreaks had been associated with any of the new BA genotype [43].

In 2009–10, a delay in RSV infections was observed. We attribute this delay to the emergence of the H1N1pdm09 influenza virus. Indeed, a similar delay in RSV infection was reported in other places in Europe [44]–[46] and was also observed with regard to other respiratory viruses, such as seasonal influenza and hMPV [47].

In summary, we demonstrate here that major changes in RSV epidemiology have occurred in the last 3–4 years in Israel. It will be interesting to investigate whether these changes also occurred in other parts of the world and whether the changes observed in RSV epidemiology will have clinical manifestations in the future.
